# L-arginine and aged garlic extract for the prevention of migraine: a study protocol for a randomised, double-blind, placebo-controlled, phase-II trial (LARGE trial)

**DOI:** 10.1186/s12883-023-03149-y

**Published:** 2023-03-27

**Authors:** Devahuti R. Chaliha, Mauro Vaccarezza, Emily Corti, Ryusuke Takechi, Satvinder S. Dhaliwal, Peter Drummond, Eric Visser, Fred K. Chen, Jason Charng, Virginie Lam, John C.L. Mamo

**Affiliations:** 1 Curtin Health Innovation Research Institute, Faculty of Health Sciences, Curtin University, Bentley, Australia; 2 School of Population Health, Faculty of Health Sciences, Curtin University, Bentley, Australia; 3 Curtin Medical School, Faculty of Health Sciences, Curtin University, Bentley, Australia; 4 Duke-NUS Medical School, National University of Singapore, Queenstown, Singapore; 5 Institute for Research in Molecular Medicine (INFORMM), Universiti Sains Malaysia, Pulau Pinang, Malaysia; 6 Singapore University of Social Sciences, 463 Clementi Road, Clementi, 599494 Singapore; 7 College of Science, Health, Engineering and Education (SHEE), Murdoch University, Murdoch, WA Australia; 8 School of Medicine, University of Notre Dame, Fremantle, Australia; 9 Centre for Ophthalmology and Visual Science (incorporating Lions Eye Institute), The University of Western Australia, Perth, WA Australia; 10 Ophthalmology, Department of Surgery, The University of Melbourne, East Melbourne, VIC Melbourne, Australia; 11 Department of Optometry, School of Allied Health, The University of Western Australia, Perth, WA Australia

**Keywords:** Aged garlic extract, L-arginine, Chronic frequent episodic migraine, Photosensitivity, Randomised controlled trial, Retinal imaging, Vascular tone.

## Abstract

**Background:**

Migraine is a common and distressing neurological condition characterised by recurrent throbbing headaches, nausea and heightened sensitivity to light and sound. Accumulating evidence suggests that cerebral arteries dilate during migraine, causing distal microvessels to constrict, which could activate nociceptors and cause onset of headache pain. If so, preventing or attenuating chronic microvascular constriction, and promoting a dilatory phenotype, may reduce frequency and/or severity of migraines. The primary aim of the L-Arginine and Aged Garlic Extract (LARGE) trial is to investigate whether oral treatment with dietary nutraceuticals, L-arginine and aged garlic extract (AGE), both systemic vasodilatory agents, will alleviate migraine frequency, duration and severity in adults with chronic frequent episodic migraines.

**Methods:**

The study is a randomised double-blind placebo-controlled phase-II single-site clinical trial conducted in Perth, Australia. The target sample is to recruit 240 participants diagnosed with chronic frequent episodic migraines between 18 and 80 years of age. Participants will be randomised to one of four treatment groups for 14 weeks (placebo induction for 2 weeks, followed by 12 weeks on one of the respective treatment arms): placebo, L-arginine, AGE, or a combination of L-arginine and AGE. The doses of L-arginine and AGE are 1.5 g and 1 g daily, respectively. The primary outcome is to assess migraine response using change in migraine frequency and intensity between baseline and 12 weeks. Secondary outcomes include the impact of L-arginine and/or AGE on photosensitivity, retinal vessel changes, and blood biomarker concentrations of vascular tone, following a 12-week intervention.

**Discussion:**

The protocol describes the oral administration of 2 nutraceutical-based interventions as possible prophylactic treatments for chronic frequent episodic migraines, with potential for direct clinical translation of outcomes. Potential limitations of the study include the fixed-dose design of each treatment arm and that in vivo neuroimaging methods, such as magnetic resonance imaging (MRI), will not be conducted to determine putative cerebro-vasodilatory changes to coincide with the outcome measures. Dose-response studies may be indicated.

**Trial registration:**

The trial was retrospectively registered with the Australian New Zealand Clinical Trials Registry ACTRN12621001476820 (Universal Trial Number: U1111-1268-1117) on 04/08/2021. This is protocol version 1, submitted on 25/11/2022.

**Supplementary Information:**

The online version contains supplementary material available at 10.1186/s12883-023-03149-y.

## Background

Migraine is one of the most common and disabling neurovascular disorders, affecting more than one billion people worldwide [1]. Considered the third most costly neurological disorder to treat, the socio-economic impacts of migraines are substantial [2, 3]. The understanding of the pathophysiology and aetiology of migraines has increased substantially over the past few decades, with emergence of new therapeutic approaches. However, appropriate access and use of current therapeutic options are scant, as migraine treatments are often related to adverse effects, and are unavailable to many patients due to strict prescribing guidelines [4–6].

The prevailing dogma of migraine suggests that vasodilatation of large cerebral arteries contributes to pain associated with migraines. We provide an alternate hypothesis for the pathophysiology of vascular-based migraines, whereby stress-induced heightened sympathetic tone results in a chronically constrictive cerebral microvascular phenotype [7]. Reduced central parenchymal blood flow could then activate nociceptors and cause headache onset. Thus, we put forward the hypothesis that prevention or attenuation of chronic microvascular constriction reduces the frequency or severity of vascular-mediated headaches. There are numerous limitations with current migraine medications, including medication overuse that can paradoxically induce headache [8, 9], many are vasoconstrictive which is a contraindication for patients with cardiovascular complications [10–12], invasive subcutaneous administration, high costs, and regulatory restrictions [11, 13]. Accordingly, benign vasodilating alternatives to prevent and treat patients with vascular-induced headaches need to be urgently considered.

L-arginine (2-amino-5-guanidinopentanoic acid, arginine) and aged garlic extract (AGE) are two oral nutraceutical agents that have been used to improve several cardiovascular disease risk factors via vasodilatory mechanisms [14–21]. However, neither have been investigated as a prophylactic option to treat chronic frequent episodic migraines. Briefly, through enhanced biosynthesis of endothelial nitric oxide (L-arginine) or increasing systemic vasodilatory prostaglandins (AGE) amongst other anti-inflammatory and antioxidant properties, L-arginine and AGE are likely to be efficacious in attenuating migraine severity/frequency induced by cerebral microvascular constriction. A significant advantage of these two nutraceuticals over current conventional migraine drug treatments is that the safety profile of both agents has been widely documented and well-tolerated in the context of cardiovascular indications with minimal side effects.

Here, we detail the study protocol for the ‘L-Arginine and Aged Garlic Extract (LARGE) for the Prevention of Migraine’ trial. The study is a 12-week, phase-II, single-site, randomised, double-blind, parallel-group-design, placebo-controlled trial, investigating the efficacy of either oral L-arginine, AGE or a combination of these nutraceuticals in adults with chronic frequent episodic migraines. Key outcome measures include frequency of migraines attacks and intensity of headache pain. Additional exploratory outcome measures include changes in photosensitivity, retinal vascular diameter, and blood biomarkers of vascular tone.

## Objectives

We are trying L-arginine and AGE for the first time for headaches, based on our proposed underlying mechanism and these nutraceuticals’ vascular applications in the past. The rationale for this study is that migraine may be caused by sympathetically induced and/or compensatory-to-vasodilation capillary constriction, leading to insufficient blood flow in the brain relative to increased energy demands in times of stress, and the triggering of trigeminal nerve endings via large-vessel dilation. We are investigating whether the known capillary-dilating agents, L-arginine and/or aged garlic extract, prevent the capillary constriction we propose underlies the headache. Therefore, our primary measure will be alleviation in migraine frequency and/or severity via self-reported subjective questionnaires; and our secondary measures will determine expansion of brain capillaries via blood biomarkers of systemic vascular tone and via retinal optical coherence tomography angiography. An additional secondary measure will be a photosensitive test using a brightening light source, to determine difference in this major migraine symptom, also before and after treatment.

The primary objective of this study is to evaluate the impact of oral 1.5 g/day L-arginine, 1 g/day AGE, or a combination of the two nutraceutical doses, on reducing migraine frequency and severity, compared with placebo, in adults with chronic frequent episodic migraines, over a 12-week intervention period. The secondary objectives are to measure the independent and combined effects of each intervention on physiological vasodilation in the retinal vessels, onset of photosensitivity, blood biochemical markers of vascular tone, and to evaluate the impact on quality of life, in adults with chronic frequent episodic migraines.

## Methods/Design

The methods reporting of this study follow the recommendations of the Standard Protocol Items: Recommendations for Interventional Trials Statement [22].

### Trial design

This is a single-site, randomised, double-blind, parallel-group-design, placebo-controlled, phase-II clinical trial on the efficacy, safety and tolerability of oral L-arginine and/or AGE in 18-80-year-old individuals with chronic frequent episodic migraines, based in Perth, Western Australia (WA). Study participants and members of the research team will be blinded to randomisation of the intervention treatments.

**Fig. 1:**
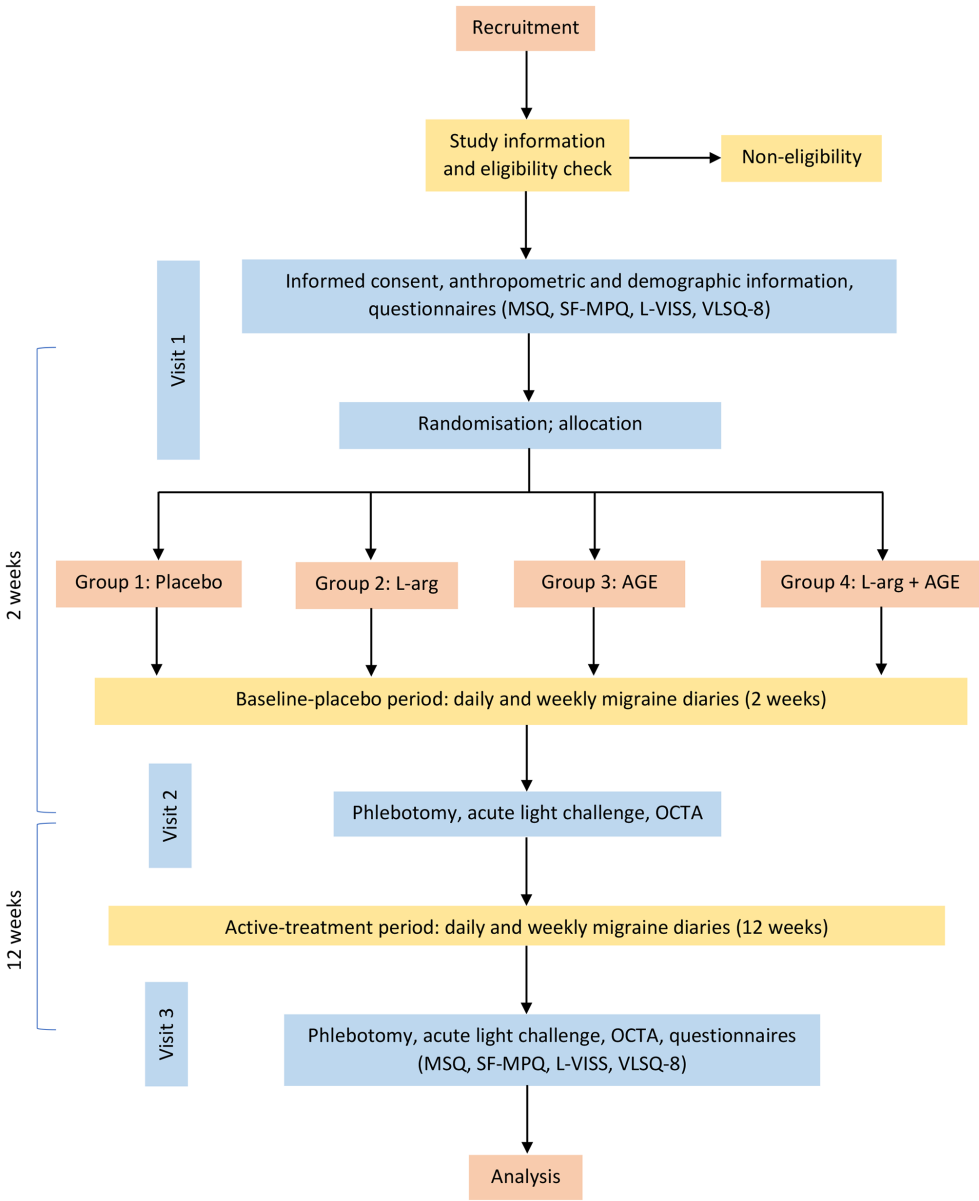
Overview of flow of participants. L-arg = L-arginine; AGE = aged garlic extract; OCTA = optical coherence tomography angiography. Participant self-report long-term questionnaires consist of the Migraine-Specific Quality-of-life Questionnaire (MSQ), Short-Form McGill Pain Questionnaire (SF-MPQ), Leiden Visual Sensitivity Scale (L-VISS), and Visual Light Sensitivity Questionnaire – 8 (VLSQ-8). Migraine diaries are completed daily and weekly over the 2 and 12 weeks between visits

The maximum study duration is 14 weeks with a 2-week placebo run-in followed by a treatment period of 12 weeks. Eligible participants will be randomised to one of four parallel treatment groups in a 1:1:1:1 ratio, using permuted block randomisation: placebo, L-arginine, AGE, and combined L-arginine and AGE. Factoring for up to 20% drop-out rate, we aim to recruit 240 participants with up to 60 individuals randomised to each of the four treatment groups. Figure 1 shows an overview of the study design for the LARGE trial. The trial is registered through the Australian New Zealand Clinical Trials Registry (ACTRN12621001476820).

### Study setting and recruitment

The single-site trial will be based in Perth (WA). Study visits will be completed at the Sarich Neuroscience Research Institute (SNRI), Nedlands (WA), with retinal imaging conducted at the Lions Eye Institute (LEI), Nedlands (WA) (adjacent to SNRI). To reach the targeted sample size, potential participants will be recruited through advertising and editorials on social media, print, radio and/or television; correspondence and promotions via not-for-profit organisations and research partners; and website content.

Prospective participants will be informed that participation is voluntary, and that withdrawal of consent is possible at any time without having to provide justification. All relevant information will be disclosed during the consent procedure with the opportunity to clarify questions with the investigator team. Enrolment is only possible after written informed consent is obtained (see Appendix 1 for participant Information Sheet and Consent Form).

### Eligibility criteria

The eligibility criteria for the participants in this study are listed in Table 1 below. The inclusion criteria must be fulfilled, and the exclusion criteria must not apply. Eligibility will be verified by delegated research personnel at the inclusion/consent visit.

**Table 1:** LARGE trial inclusion and exclusion criteria

Inclusion criteria	Exclusion criteria
1. Migraine (with or without aura) diagnosed at least 1 year ago 2. Migraine onset occurred before 50 years of age 3. Adult males and females, aged 18–80 years (inclusive) at screening 4. 2–6 migraine episodes and fewer than 6 ‘other’ headache types per month, averaged over the previous 3 months 5. Able to differentiate between migraine and ‘other’ headache types 6. Able to commit to taking oral capsules once a day for 14 weeks 7. Able to fill out paper daily migraine diary over 14 weeks	1. Taking medications/drugs that affect vascular tone or blood pressure 2. Continuous residual headaches 3. Headache disorder or facial pain disorder other than migraine 4. Change in migraine treatment in previous 3 months 5. Taking > 2 migraine-prevention drugs 6. L-arginine tablets or aged garlic extracts in previous 3 months 7. Possibility of pregnancy or breastfeeding during study 8. Adverse reactions to food containing L-arginine or garlic 9. Clinical reports of kidney/liver dysfunction 10. Known risks of bleeding or coagulopathy, or currently on blood-thinning medications 11. Cancer, haemochromatosis or diabetes 12. Severe psychiatric disorder or other neurological disorders 13. Acute/chronic pain disorders, infections, cardiovascular or cerebrovascular disease 14. Poorly controlled depression or anxiety 15. Antipsychotic or antidepressant medications/drugs over past 3 months 16. Low blood pressure or complications arising from it 17. Diagnoses other than migraine as primary cause of headaches 18. Changes in digestive system over past few months 19. Headaches cause fainting or other medical emergencies 20. Substance abuse/dependence/addiction in previous 3 months 21. Procedure or injection to treat migraine over past 3 months 22. Implanted or external nerve stimulator to treat migraines 23. Pain medications on most days a week for headaches 24. Opioid medications each day for chronic pain 25. History of overusing medications 26. History of eye pathology, surface disorder, surgery (except cataract extraction), or injury 27. Difficulty seeing clearly (with glasses or contact lenses, if required) 28. History of diabetes, hypertension, collagen vascular disease, vasculitis, or renal disease/failure 29. Smoke > 1 pack of cigarettes a day 30. Disorders of the optic nerve or retina 31. Reasons that retinae may not be clearly viewed 32. Poor image perception due to cataract or unstable fixation 33. Anaemia or iron deficiency 34. Participation in concurrent research trial

### Study intervention

#### Intervention description and adherence

This study consists of 2 phases, including: (a) a 2-week placebo run-in phase (week − 2 to 0); (b) a 12-week treatment phase (day 0 to day 84). The Diener and Tassorelli guidelines suggest that a 4-8-week screening phase is ideal to ensure diagnostic migraine criteria are met for both episodic and chronic migraines [23, 24]. However, since participants could benefit from 3 months’ treatment without a high attrition rate, 4–8 weeks’ placebo would already take up half the investigation time for each participant. We are relying on participant numbers to compensate to power the study, and we have already ensured diagnostic criteria are met, as all participants must have confirmation from a physician or neurologist that they have migraine. We have included the extensive list of eligibility criteria, including frequency of migraines participants must have to be included in the trial. Participants are asked to report any adverse effects that arise from the treatment, at absolutely any point during their time in the trial. They are contacted one week after each of visits 1 and 2 to reinforce this and to check treatment compliance. Leftover capsules are counted at the end of each participant’s 2 and 12 weeks, to check placebo and verum treatment compliance, respectively.

Once individuals are deemed eligible according to the inclusion and exclusion criteria listed in Table 1, participants will be asked to provide written consent and will be enrolled into the trial. At the consent visit (visit 1), participants will be asked to provide basic demographic information, complete medical and migraine history (i.e., duration of migraine, age of onset, monthly migraine frequency), current medication, menstrual status if applicable, and medical history. Baseline demographic and anthropometric measures were adapted from the National Institute of Neurological Disorders and Stroke Common Data Elements (www.commondataelements.ninds.nih.gov) and the PhenX toolkit (https://www.phenxtoolkit.org/), respectively. During this visit, participants will be instructed on how to fill out the daily and weekly migraine diaries. All participants are required to undergo a 2-week placebo run-in and are required to take oral placebo capsules for 2 weeks (5 capsules per day). Thereafter, at visit 2 (baseline), a 12-week supply of the respective randomised treatment will be dispensed to each participant; 5 capsules a day for 12 weeks (placebo; L-arginine; AGE; combination of L-arginine and AGE). Participants are contacted one week following commencement of treatment, to discuss any potential adverse events and treatment compliance.

Each participant will be provided with a paper-based diary to improve adherence of all groups (see Appendices 2 and 3). Treatment efficacy in terms of headache pain, severity and frequency will be evaluated by a standardised daily migraine diary (Appendix 2) and a summarising weekly migraine diary to track clearer long-term progress (Appendix 3). A capsule information sheet is dispensed with the intervention capsules to provide details instructions on missed doses where relevant. Similar to the placebo run-in, participants will be required to return capsule bottles at the end of the 12-week intervention to determine adherence to treatment. Poorly compliant participants will be excluded from subsequent analyses.

Visit 3 will be an exact replica of visit 2, with the difference that no further capsules/diaries will be given, and the visit-1 long-term questionnaires will have been filled out again to compare with the results of the visit-1 ones. Blood biomarkers, photosensitivity tests and retinal scans will be compared between visits 2 and 3 to test differences due to treatment over the 12 weeks.

#### Supply of intervention

Trial interventions (including placebo) are provided as identical capsules bottled in participant-ID-labelled containers by a GMP-certified compounding pharmacy, Oxford Compounding (North Perth, Australia). The placebo capsules are comprised of microcrystalline cellulose with no active ingredients. No dose modification of the trial intervention is permitted. All capsules are lightly scented with garlic to avoid potential bias.

#### Discontinuing or modifying allocated interventions

If a participant decides to withdraw from the project, participants are asked to notify a member of the research team. If a participant withdraws consent during the research project, relevant project team members will not collect additional personal information from the participant. However, personal information already collected will be retained to ensure that the results of the research project can be assessed and to comply with the law. Participants will be made aware that data collected by the investigator team up to the time the participant withdraws will form part of the research project results.

#### Relevant concomitant care during the trial

Apart from the medications in the exclusion criteria above, participants will be able to continue their regular medically prescribed regimen. Participants will be encouraged to inform their general practitioners (GPs) that they are participating in this study. Participants should not partake in other clinical trials while participating in this study.

### Study procedures

Table 2, schedule of assessments, details the overall schedule of the trial.

**Table 2:** LARGE trial schedule of assessments

	STUDY PERIOD					
Visit	Screening /enrolment	Visit 1 (informed consent)		Visit 2		Visit 3 (EoS)
TIMEPOINT	-t_1_ (1 day)	0 (1 day)	t_1_ (2 weeks)	t_2_ (1 day)	t_3_ (12 weeks)	t_4_ (1 day)
**ENROLMENT**:						
**Eligibility screen**	X					
**Informed consent**		X				
**Participant demographics**		X				
**Participant anthropometric measures**		X				
**Group allocation**		X				
**INTERVENTIONS**:						
**Placebo**			X		X	
**L-arg**					X	
**AGE**					X	
**L-arg + AGE**					X	
**ASSESSMENTS**:						
**Daily and weekly migraine diaries**			X		X	
**MSQ**		X				X
**SF-MPQ**		X				X
**L-VISS**		X				X
**VLSQ-8**		X				X
**OCTA**				X		X
**Acute light challenge**				X		X
**Blood markers**				X		X

L-arg = l-arginine; AGE = aged garlic extract; t = timepoint; MSQ = Migraine-Specific Quality-of-life Questionnaire; SF-MPQ = Short-Form McGill Pain Questionnaire; L-VISS = Leiden Visual Sensitivity Scale; VLSQ-8 = Visual Light Sensitivity Questionnaire – 8; OCTA = optical coherence tomography angiography

### Primary and secondary outcomes

#### Primary outcomes

The primary objective of the LARGE trial is to evaluate the effectiveness of oral supplementation of L-arginine, AGE or the combined treatment of oral L-arginine and AGE, in reducing the severity and frequency of migraine attacks in chronic frequent episodic migraines in adults. By chronic frequent episodic migraineurs, we include both episodic and chronic, as well as uncertain: from published official guidelines, some clinical trial participants may not fit neatly into having episodic or chronic migraines, and/or into with or without aura, but they are nonetheless included [25]. In accordance with the International Headache Society guidelines for RCTs in preventative treatment of episodic migraines in adults [23], the differences in the mean number of migraine episodes per month, between baseline (visit 2) and end of study (EoS; 12 weeks treatment, visit 3), will be determined as the primary outcome measure assessed by a standardised daily migraine diary (Appendix 3). Migraine frequency will be measured not just within each migraine day, but the number of migraine occurrence days over the weeks. From the daily migraine diaries, treatment efficacy will be evaluated by the mean change in outcome measures between baseline and EoS within each group, and thereafter compared with changes between each intervention group. Primary outcomes collated from the diaries include: (a) mean number of migraine episodes per month (migraine frequency); (b) mean number of migraine days per month; (c) mean number of moderate-or-severe migraine days per month; (d) mean duration of each migraine episode (hours) per month; (e) mean number of acute migraine medication doses per month; and (f) number and type of self-reported adverse events per month. We will not measure migraine episode duration, because the participant is able to use an abortive drug at any point, and sleep intervals can disrupt countability of each episode.

#### Secondary outcomes

Secondary outcomes will comprise the changes in acute photosensitivity, quality of life (QoL), retinal vessel changes, and blood biomarkers of vascular tone, from baseline to EoS.

To explore the effects of each intervention arm relative to placebo on photosensitivity, a visual discomfort threshold will be determined via an acute light challenge on the second and third visits. Briefly, adapted from Woodhouse and Drummond (1993) [26], to determine the visual discomfort threshold, participants were subjected to an increased brightness of an LED panel facing them 200 cm away by 1% every 4 s. The percentage brightness of the Nanlite Mixpanel 150 RGBWW 150 W LED Panel (emission range: 0–11,320 lx) at which the participant expresses discomfort and requests no further increase in brightness will be recorded at both study visits and compared to determine differences if any, in visual sensitivity.

The self-reported Migraine-Specific Quality-of-life Questionnaire (version 2.1) (MSQ) [27, 28] and Short-Form McGill Pain Questionnaire (SF-MPQ) [29] will be completed by each participant at baseline and EoS. The MSQ is utilised to assess the change, before and after treatment, in each of the treatment groups in participants’ day-to-day functioning about impeding activity, preventing activity, and emotional feelings, that are usually affected by migraines. The SF-MPQ is used to evaluate weekly differences in pain intensity reported by each of the participants. The Leiden Visual Sensitivity Questionnaire (L-VISS) measures the impact of light and pattern sensitivity on daily functioning (“visual allodynia”) [30], and the Visual Light Sensitivity Questionnaire (VLSQ-8) measures the presence and severity of photosensitivity symptoms [31].

A number of recent studies have implicated lower vessel densities in the macula and optic nerve, as well as reduced thicknesses of the choroid and retina, in adults with migraines compared to controls without migraines [30–42]. Thus, an additional exploratory outcome will comprise the changes in retinal vessel density and the foveal avascular zone area measured via optical coherence tomography angiography (OCTA), with the AngioVue Imaging System (RTVue XR Avanti, Optovue Inc., Fremont, CA, USA), at baseline and 12-weeks post intervention, as a surrogate measure of cerebral vascular changes.

Moreover, blood biomarkers of vascular tone will be analysed in samples collected at baseline and EoS. Systemic concentrations of asymmetric dimethylarginine (ADMA) and symmetric dimethylarginine (SDMA), nitrates and nitrites, and endothelin-1, will be analysed in fasted plasma samples. Among our proposed panel of biomarkers, the antagonism between vasodilatory NO and vasoconstrictive endothelin-1 allows the assessment of potential changes in vascular tone [43]. Nitrates and nitrites are more stable derivatives of reactive NO in the bloodstream [44], and thus were chosen as a proxy measure of dilation of the vessels supplying the brain. ADMA and SDMA, additionally, would be valuable measures of interictal propensity to migraine, as higher levels of both vasoconstrictive molecules would reflect inhibition of vasodilatory NO production from L-arginine [43].

### Statistical analysis

#### Sample size and statistical power

We propose an RCT with a factorial treatment structure. This design is efficient and allows the estimation of both the main effects of L-arginine and AGE, and the interaction between L-arginine and AGE. This design with two factors at 2 and 2 levels has 4 arms/groups (treatment combinations). A total of 240 individuals are required to provide 60 subjects per group. This design achieves 87% power when an F-test is used to test factor L-arginine at a 5% significance level with the effect size of 0.2; achieves 87% power when an F-test is used to test factor AGE at a 5% significance level with the effect size of 0.2; and achieves 87% power when an F-test is used to test the L-arginine*AGE interaction at a 5% significance level with the effect size of 0.2. Cohen’s Effect Size Table [45] provides the interpretation of effect sizes as: Small: 0.1; Medium: 0.25; Large: 0.40. Our research study can therefore detect between small and medium effect sizes between each of the main effects and interactions. An estimated effect size of 0.2, used in the sample size calculation for testing the main effects and interaction, was obtained from our preliminary data. Assuming a dropout rate of 20%, a total of 300 participants will be initially recruited into the study, with 75 participants in each of the 4 treatment combinations.

#### Statistical methods and exploratory analysis

We will analyse the data using IBM SPSS® Statistics for Windows, Version 24.0 (IBM Corp., Armonk NY, USA and Stata Version 17 (StataCorp, 4905 Lakeway Dr., College Station, TX 77,845, USA)). P-values less than 0.05 will be considered statistically significant. As the proposed study has 4 treatment arms, multiplicity adjustments will be utilised in the statistical analysis of data. The family-wise error rate is set to be 0.05 to safeguard against the increase in the probability Type-I error due to the number of multiple comparisons.

Continuous data will be reported as a group mean ± standard deviation (SD), and categorical data as count (percentage). Data from continuous variable outcomes will be analysed using an Analyse of Covariance procedure as a General Linear Model, with the baseline values as covariates. Post-hoc comparison of means between the 4 treatment combinations will only be conducted if the interaction between sex and treatment is statistically significant. We will take differences between treatment groups as mean differences with associated 95% confidence intervals. We will analyse categorical outcome variables using a Multivariable Logistic Regression Model, with the baseline values as covariates. Treatment effects will be expressed as odds ratios with associated 95% confidence intervals. Residuals from each of the analyses will be inspected to ensure validity of the statistical procedures utilised. Appropriate graphical displays will be used to demonstrate and communicate the main effects and interactive effects of the treatment groups.

Mixed models, which explicitly account for the correlations between repeated measures within each subject, will be utilised in the statistical analysis of the data. The mixed model will assess the extent to which the subject’s trajectory is influenced both by the effect of an intervention and by baseline characteristics. Plots illustrating the outcome trajectories of individual study subjects over time will be used to determine whether the observed data patterns appear consistent with model assumptions.

### Allocation

Randomisation occurs on visit 1, in that the coded capsule bottle is picked up randomly and allocated to this next participant scheduled. One week after this first visit, they are texted to check progress on the capsules. We are using block randomisation: eligible subjects will be computer-randomised in blocks of 8, to one of the 4 treatment groups (placebo control, L-arginine, aged garlic extract (AGE), and L-arginine + AGE). Each participant will be randomised by participant ID, as this should preclude group allocation by order of study entry, as per the IHS guidelines [24, 25]. We will do this as the participants enter the trial, whereby the statistician will then have access to the list on the Research Data Capture (REDCap) tool where we will be entering all participant details and responses.

#### Implementation

The randomisation schedule is sent to an independent pharmacist (Oxford Pharmaceuticals) who will then dispense trial intervention in blank packaging with only the participant ID displayed. The investigators will receive this by post and will supply each participant with the treatment pack on visits 1 and 2 (placebo for 2 weeks, then randomised treatment for 12 weeks).

### Blinding

Although participants will be blinded to treatment allocation, we will inform them of the general nature and composition of the treatment prior to their participating. The investigator team will be blinded as to which treatment we are giving which participant, during the 12 intervention weeks. Only the statistician and pharmacist will not be blinded as to which participant receives which treatment, as they will be handling the randomisation schedules linking identification numbers to participant names. In case of emergency (an adverse event sustained by the participant, which requires medical treatment to know which study treatment has been given), the investigator will break the seal and be unblinded as to which treatment the participant has received. This will immediately be reported back to the investigator team and the HREC. The participant always has the option of dropping out of the trial.

### Data collection and management

Data will be collected by delegated personnel from the research team. We will compile de-identified data into monthly data sets, using Microsoft Excel® Version 15.0 (Microsoft Corporation, Redmond WA, USA). For OCTA, photosensitivity and plasma biomarkers, the computed results will be copied via a password-protected USB stick to the university research drive. Identifiable data will be sorted by date in separate folders according to each questionnaire and stored in a locked filing cabinet with access restricted to the investigator team. All other data will be de-identified to ensure confidentiality of participant data. Data will be stored on a password-protected web-enabled clinical-trial-data electronic management system (REDCap) located in an ISO27001-compliant facility at Curtin University. Participants’ study information will not be released outside of the study without the written permission of the participant. All data will be securely archived as per the Institute’s data policy for a minimum of 15 years. A formal data-monitoring committee was not required for ethics approval. The principal investigator will terminate the trial if appropriate, for example due to unforeseen adverse events associated with the intervention.

## Discussion

Our trial proposal stems from the positive action on vasodilatation exerted by L-arginine and AGE in the cardiovascular setting [7, 15, 46]. Although new treatments and preventions are being explored for migraine treatment [47, 48], L-arginine and AGE have not been clinically considered.

L-arginine is an amino acid that is involved in the production of nitric oxide, which helps to relax blood vessels and improve blood flow. Aged garlic extract is a supplement derived from garlic that is aged for up to 20 months [49, 50]. It is believed to have antioxidant and anti-inflammatory properties, and from some studies, we hope that, similar to L-arginine, it may also be effective in reducing the frequency of migraines [51, 52]. However, more research is needed to confirm these findings and understand how aged garlic extract may work to prevent migraines.

We surmise that these agents should be investigated in future larger randomised controlled trials for prevention of migraines induced by cerebral microvascular constriction. To assess and confirm regional vasoconstriction of cerebral arterioles and capillaries, we recommend imaging of the cerebral microvasculature directly using functional imaging techniques [53, 54].

It is important to note that while these supplements may have potential benefits, in principle, they should not be used as a replacement for conventional migraine treatments. Additionally, it is also worth noting that both L-arginine and aged garlic extract can interact with certain medications, so it is important to talk to one’s doctor before taking them if one is currently on any prescription medications.

One important limitation is that currently, no studies have investigated the effects of L-arginine and AGE in the context of gender or ageing. These variables are established risk factors for headaches, concomitant with decreased endogenous biosynthesis of NO [55–57]. All previous studies show an alleviating effect of both L-arginine and AGE in all 3 aspects: decreased inflammation and oxidative damage markers, decreased triglycerides and low-density lipoproteins, increased high-density lipoproteins, decreased atherosclerosis, and decreased thrombotic events [7]. The fact that neither L-arginine nor AGE have been tried in randomised controlled trials, specifically for the prevention of migraine, is a current shortcoming. Our study is amending this issue, and it is of potential clinical value, even considering the study population (appropriate numbers, subject intake not limited by age in principle) that we are going to assess.

The value of our proposal is also based on the fact that accessibility, optimal usage and costs are still unresolved issues for new drugs against migraine [47, 58], and the molecules that we are testing are affordable and already used in humans, with minimal side effects. Other strengths of our proposal include the randomised placebo-controlled trial structure, the use of both males and females, and a wider age range to include above-65-year-olds – which allows better assessment of potential safety issues in more vulnerable (until age 80) populations. Also, > 60-year-olds are different in symptom onset [59], so including this subgroup would make the results more generalisable. Given the stringent eligibility criteria, we realised that there was no reason not to include individuals older than 65 as specified in Diener and Tassorelli [23, 24], especially as a significant safety issue was not present. Older than 80 years old may present comorbidity-related confounders.

Since older subjects also tend to use polypharmacy, any unforeseen interaction effects (despite having checked for potential contraindicated drug interactions in the eligibility criteria) should come to light. Our treatment is an oral preventive which is non-invasive, and would hopefully preclude the need for combination therapy in treatment-refractive populations with a vasoconstrictive migraine aetiology. Combination therapy generally increases risk of adverse effects, and it is difficult to ascertain which drug each effect may come from and may need to be withheld [47]. We use both subjective (diaries and questionnaires) and objective measures (vascular tone, photosensitivity and blood markers) to track migraine progress longitudinally in each participant, with a placebo wash-in to further increase reliability of results. The nutraceutical agents are mildly vasodilatory, hence are not contraindicated for patients with pre-existing vasoconstrictive issues, nor should they pose a danger to patients with hypotension (we also measure blood pressure at each visit). The 2 × 2 group schedule allows a placebo control, as well as checking for interaction effects when both nutraceuticals are simultaneously given. Reported well-tolerated doses of L-arginine and AGE, considered for cardiovascular disease risk, serve as a useful guide to explore the putative efficacy of L-arginine and AGE for prevention/treatment of vascular-associated migraine.

### Patient and public involvement

No participants were involved in the design of this study.

### Research ethics approval and results dissemination

The LARGE trial has been approved by the Curtin University Human Research Ethics Committee (HREC) (Approval number: HRE2020-0466; Version 4; 16th August 2021). The trial is registered with the WHO Trial Registration Data Set. The Universal Trial Number is U1111-1268-1117. Written consent will be obtained from all participants prior to commencing their participation in the trial. The consent form is included as Appendix 1. Any protocol amendments will be submitted straight to ethics for approval before implementation, and the trial registry accordingly updated.

The results of the study will be disseminated in peer-reviewed publications and presented at key national and international conferences and local stakeholder events.

### Trial status

Participant recruitment commenced in May 2021, and follow-up analyses are expected to complete in 2023.

## Electronic supplementary material

Below is the link to the electronic supplementary material.

Supplementary Material 1

## Data Availability

All investigators will have access to the final dataset. The corresponding author (JM) should be contacted to request data from this study.

## Abbreviations

L-arg: L-arginine
AGE: Aged garlic extract
CHIRI: Curtin Health Innovation Research Institute
GP: General practitioner
HREC: Human Research Ethics Committee
HIS: International Headache Society
LED: Light-emitting diode
LEI: Lions Eye Institute
L-VISS: Leiden Visual Sensitivity Scale
MSQ: Migraine-Specific Quality-of-life Questionnaire
OCTA: Optical coherence tomography angiography
PGE-5: Phosphodiesterase type 5
REDCap: Research Data Capture
SF-MPQ: Short-Form McGill Pain Questionnaire
SNRI: Sarich Neuroscience Research Institute
VLSQ-8: Visual Light Sensitivity Questionnaire – 8
