# Supplementary material for: L-arginine and aged garlic extract for the prevention of migraine: a study protocol for a randomised, double-blind, placebo-controlled, phase-II trial (LARGE trial)
Source: BMC Neurol. 2023 Mar 27;23:122. doi: 10.1186/s12883-023-03149-y (PMC10041759; doi:10.1186/s12883-023-03149-y)
Supplement: Supplementary file 1 — Supplementary Material 1 [file 12883_2023_3149_MOESM1_ESM.docx]

**APPENDICES**

**Appendix 1**

1. **Information Sheet**

**Migraine Treatment Research Project**

**Participant Information Sheet**

You have been asked to participate in this study involving a new treatment for migraines. Please read this document carefully and ask any questions you wish. Knowing what is involved will help you decide if you want to take part in this research. Before deciding whether to take part, you might want to talk about it with a relative, friend or your local doctor. Participation in this research is voluntary. Do not sign this informed consent form unless you fully understand the nature of the study and any possible side effects. There will be ample opportunity for you to meet the investigators and have the study procedures and protocol fully explained to you.

**Background information**

Migraine is a very common and distressing neurological condition that causes symptoms such as recurrent throbbing headaches, nausea and disturbed vision. Migraines are caused by the interaction between the brain and blood vessels of the brain. Migraines are conventionally believed to occur because of the dilation (opening) of the large vessels in the brain, which leads to activation of pain receptors in the brain which causes the common symptoms of migraines. The medications currently offered do not seem to address the main cause of migraines, and have many adverse effects.

Recent research findings suggest that migraines may occur due to the constriction (tightening) of the smaller vessels of the brain, that leads to oxygen and nutrient insufficiency, causing vascular headaches; however, there are questions that still need to be answered. There are certain dietary compounds that can increase the dilation of smaller blood vessels, and our research project will investigate if these compounds can reduce the frequency and severity of migraine symptoms. These include L-arginine and aged garlic extract. L-arginine is an amino acid commonly found in food, that helps the body build protein. It is found in meat, dairy, eggs and seeds. Aged garlic extract is derived from garlic which is a common ingredient in food. Garlic is actually known to have beneficial antioxidant, anti-thrombotic and anti-atherosclerotic properties. Neither have been commonly known to cause adverse effects when included in the human diet.

In this study, researchers are seeking participants to investigate the preventive/treatment effects of L-arginine and aged garlic extract in reducing migraines headaches both in the short- and the long-term. This study is a double-blind study. To ensure we test without bias whether each intervention is effective in reducing the severity of migraines, participants will be randomly assigned to one of four groups: group 1 (placebo); group 2 (L-arginine); group 3 (aged garlic extract); or group 4 (a combination of L-arginine and aged garlic extract). You will be randomly allocated (by computer) and have an equal chance to be in any of the groups. As this is a double-blind study, neither you nor the research team will know which group you are allocated to, in order to reduce bias throughout the treatment.

**Inclusion/exclusion criteria**

**To be eligible for this trial, you must fulfil the following inclusion criteria**:

1. Migraine (with or without aura) diagnosed at least 1 year ago
2. Migraine onset occurred before 50 years of age
3. 2-6 migraine episodes and fewer than 6 'other' headache types per month, over the last 3 months
4. Able to distinguish between migraine and 'other' headache types
5. Able to complete a daily diary about migraine experience
6. Able to commit to taking the 5 capsules a day for 14 weeks

**If you meet any of the following criteria, you will not be eligible for the study:**

1. Taking medications/drugs affecting vascular tone or blood pressure
2. Taking nitrate drugs or isoproterenol (often prescribed for angina or heart failure)
3. Taking more than two migraine-prevention drugs
4. Taking antidepressants or diuretics, herbs, supplements
5. Taking Sildafenil, Cialis, Spedra and any other PGE-5 inhibitor drugs
6. Taking drugs with potential blood-vessel effects (analgesics, decongestants, or antihistamines)
7. Already taking L-arginine or garlic supplements for 3 months before the study
8. Having headaches causing fainting or another medical emergency
9. Having chronic daily headaches, medication-overuse headaches and/or other secondary headache disorders
10. Having diagnoses other than migraine as the primary cause of headache
11. A change in migraine treatment in the 3 months prior to or during the study
12. Clinical reports of renal or liver dysfunction
13. Clinical risks associated with bleeding or coagulopathy or currently on blood-thinning medications such as warfarin/heparin therapy
14. Having any cardiovascular or neoplastic diseases
15. Having major chronic metabolic or neurologic disorders, or receiving current therapy for them
16. Being diagnosed with psychosis or bipolar affective disorder
17. Diagnosis of cancer
18. Substance abuse/dependence/addiction in the 3 months prior to or during the study
19. Having a history of diabetes, hypertension, collagen vascular disease, vasculitis, or renal disease/failure
20. Having any low-blood-pressure-related issues or Type-1 or -2 diabetes
21. Having the possibility of pregnancy or lactation
22. Being allergic to garlic or its constituents
23. Having gastric disturbances such as bloating, stomach pain, heartburn, diarrhoea, constipation, nausea or vomiting
24. Are a smoker of >1 pack a day
25. Having a history of eye pathology, surface disorder, surgery (except cataract extraction), injury
26. Not being able to see clearly (with glasses/contacts, if need be)
27. Having disorders of the optic nerve (including glaucoma) or retina
28. The significant possibility of our not being able to see the inside of your eye clearly
29. Having poor image perception due to cataract or unstable fixation

**What does the study involve?**

This study is open to adults aged between 18 and 80, who currently experience migraines. This is a randomised controlled trial, which means that study participants will be randomly placed into groups and given different treatments in order for the results from each to be compared. You will be randomly allocated to one of 4 study groups. Your participation in this study involves a considerable time commitment with a total of **3 visits** over a period of 14 weeks. The 3 visits will take approximately 30 to 60 minutes each.

Before your appointment:
Prior to your first visit, you will be sent a few questionnaires that need to be completed and brought with you to your first visit. These questionnaires will ask about your experience with migraines and the impact of migraines on your daily life. If you have not completed the questionnaires prior to your first visit, there will be time to complete the questionnaires during the visit.

Visit 1:

You will be asked to attend an appointment at the Sarich Institute (8 Verdun St, Nedlands WA 6009). During this appointment, we will discuss the study requirements and go over the inclusion and exclusion criteria for the study. If you are eligible for the study, we will measure your body weight, height, waist and hip circumferences, blood pressure and heart rate. Next, we will provide you with a treatment pack of capsules to take every day for 2 weeks. During the next 2 weeks, we ask you to take **five (5)** capsules each day, and complete a hardcopy daily and weekly migraine diary we will provide you with. This session should take approximately 30 minutes.

Visit 2:

2 weeks after Visit 1, you will be asked to attend the Sarich Institute again (8 Verdun St, Nedlands WA 6009). During this session, we will take a sample of your blood and measure your blood pressure, and you will be asked to complete a light-sensitivity task and undergo an eye (retinal) scan. For the blood collection, we will ask you to come in fasted (8-12 hours), only consuming water before we collect your blood. All blood samples will be collected by a certified phlebotomist. If we cannot collect a blood sample from you, you may be requested to visit PathWest for a blood test. In the rare event of a medical emergency, fully qualified physicians and nursing staff from the Sarich Institute will be available. The retinal imaging is completely non-invasive and pain-free (it does not even require eye drops), and it should only take 5 minutes to scan each eye. The reason for this retinal scan is that the small vessels of the eye (within the retina) are considered an extension of the tiny blood vessels of the brain, and therefore may serve as a surrogate marker of central nervous system (brain) small-vessel response to our treatment.

For the light-sensitivity task, you will be asked to sit in a dark room, and we will increase the luminosity of a light panel in front of you in a step-wise fashion until you feel the light is becoming uncomfortable, or continued exposure to the light would bring on a migraine. This may cause some discomfort; however, this will be aborted as soon as you indicate the light is uncomfortable.

Finally, you will receive your second treatment pack, comprising treatment doses to last the next 12 weeks. Again, we ask you to take the capsules and complete the migraine diary on a daily basis for the next 12 weeks. This session should last approximately 60 minutes.

Visit 3:

After 12 weeks, you will be asked to visit the Sarich Institute (8 Verdun St, Nedlands WA 6009) one last time. Prior to the appointment, you will be sent questionnaires that need to be completed prior to your appointment. These are the same questionnaires that you completed prior to visit 1. If you are unable to complete the questionnaires prior to the visit, there will be time during the visit to complete the questionnaires. Visit 3 will include the same assessments as visit 2. This session should last approximately 60 minutes.

**Possible adverse effects**

The blood collection during each visit may cause discomfort, as we will use a needle to collect the blood. Although we will try to collect the blood from different sites, it is possible that you may find the number of blood samples collected an uncomfortable experience. Therefore, it is important to inform us if this is the case and to know that you are free to withdraw from the study at any stage. For us, your comfort during the procedure is of more concern than our ability to collect both blood samples. In some people, slight bruising and tenderness may appear afterwards at the site of venepuncture. These side effects are only minor, and will return to normal in a matter of days. The volume of blood collected should not cause you any risk of becoming anaemic (approximately 6 tablespoons). Moreover, the visits have been spaced 12 weeks between each visit, so that new red blood cells will be ready to enter the circulation. However, we advise you not to donate blood during the period of study. If you need to do any routine clinical test with blood collection, this is not a problem.

L-arginine and aged garlic extract have not been known to cause any serious adverse effects. You may experience some gastric disturbances, and in rare cases, nausea and vomiting. We will contact you after the first week of the first 2-week treatment period, and after the first week of the second 12-week treatment period, to check how you are going.

**Ability to withdraw from the study**

It must be stressed that your participation in this study is entirely voluntary; you are free to withdraw from the study at any stage. It is important that you do not feel any pressure to complete the study, particularly if it is not what you had originally anticipated. In the event that you withdraw or discontinue your participation in the project, we will ask your permission to use the data you have already provided. If you do not want your data to be retained, all data relating to your participation will be destroyed. In some instances, it may be required that we retain information about any adverse events you may have experienced. You will not be identifiable from this data. If you are currently enrolled as a student at Curtin University, there will be no impact on your studies if you were to withdraw from the study.

**Benefits to the participant**

This study may not be of direct benefit to you; however, by taking part in this study, you will gain useful information about your vascular tone as a migraineur. We encourage you to let your doctor know that you are taking part in our study. Any results that are out of clinical range will be sent to your general practitioner with your consent. Your participation in this study will provide us with important data, enabling us to investigate further the role of L-arginine and aged garlic extract in treating and/or preventing vascular headaches, especially migraines.

**What happens when the research project ends?**

Your data will be stored for a minimum of 15 years after the study, then destroyed. If published studies suggest benefits are realised via the indicated treatments being trialed, we will recommend participants discuss with their primary care provider, consideration for prevention and attenuation of migraine pain frequency and intensity. You will have the option of being contacted with the results of the study.

**Confidentiality**

Any information that you provide us will be stored securely, to protect your privacy. Some of your data may include personal information such as your name, date of birth and/or a reference number. This information will be separate to your actual data (assessment scores) and your data will be de-identified. The security of standards of all personal information follows Curtin University data-management guidelines. All information will be held in secure, locked filing cabinets at Curtin University, or on a password-protected computer database held on a secure system, which allows access to authorised individuals only. It is possible that the results from this study will also be utilised in future studies on a similar topic; however, your identity will never be disclosed. All information will be strictly confidential, and any publications arising from this work will not include your name or any other identifying feature.

**Site map and parking**

Here is visitor-parking information around the Sarich Institute: <https://qeiimc.health.wa.gov.au/travel-access/parking/visitor-parking/>. Your visits will take up to an hour, and therefore will be $3.40 on the parking meter. Below is the site map, with the relevant areas circled in red. As you can see, there are 4 available carparks near the precinct.


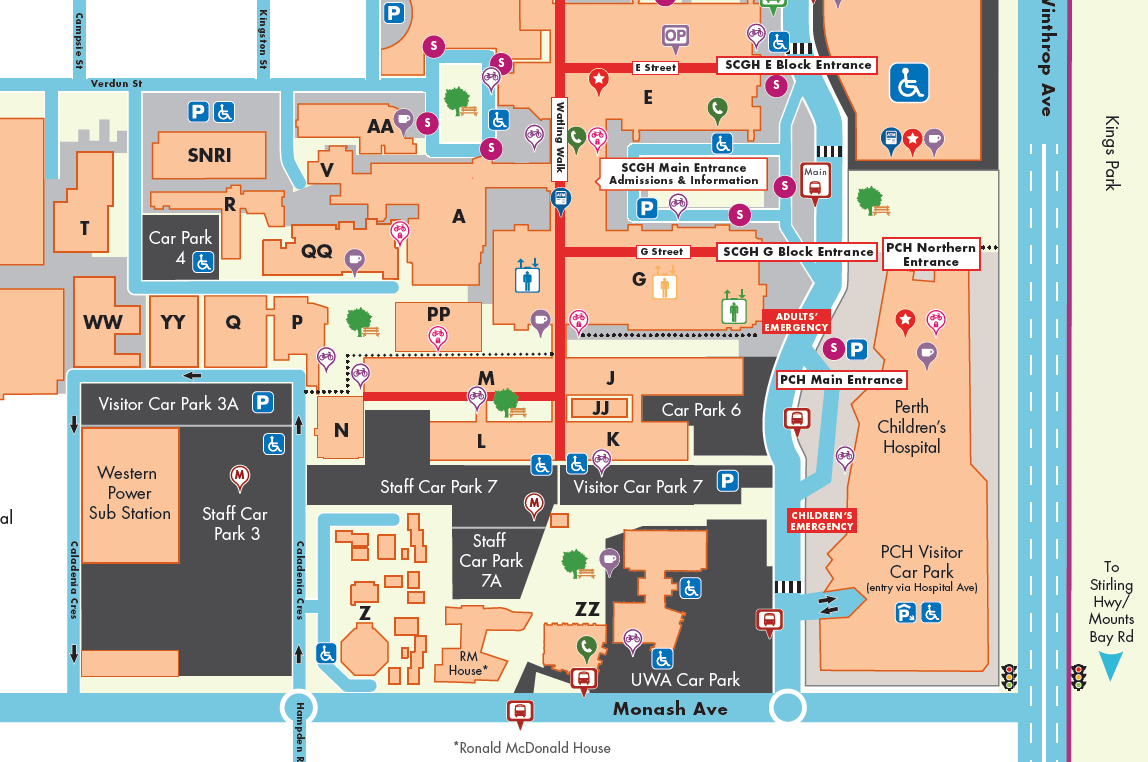


Sarich Institute

Should you get lost on the way, please don’t hesitate to call Debs on (04) 517 176 70.

**Further information**

If you have queries or require urgent contact, call Debs Chaliha on 0451717670.

If you have extensive queries or require further clarification, please contact the lead investigator:

Professor John Mamo

School of Public Health, Curtin University

Phone: (08) 9266 7232

Email: [J.Mamo@Curtin.edu.au](mailto:J.Mamo@Curtin.edu.au)

Curtin University Human Research Ethics Committee (HREC) has approved this study (HRE2020-0466). The Committee is comprised of members of the public, academics, lawyers, doctors and pastoral carers. Its main role is to protect participants. If needed, verification of approval can be obtained either by writing to the Curtin University Human Research Ethics Committee, c/- Office of Research and Development, Curtin University, GPO Box U1987, Perth, 6845, by telephoning 9266 2784, or by emailing [hrec@curtin.edu.au](mailto:hrec@curtin.edu.au).

| **HREC Project Number** | HRE2020-0466 |
| --- | --- |
| **Project Title** | Migraine Treatment Research Project (NOPAIN) |
| **Principal Investigator** | Prof. John Mamo (Director of Curtin Health Innovation Research Institute) |
| **Student Researcher** | Devahuti (Debs) Chaliha |
| **Version Number** | 3 |
| **Version Date** | 24 Feb 2021 |

- I have read the information statement version listed above, and I understand its contents.
- I believe I understand the purpose, extent and possible risks of my involvement in this project.
- I have had an opportunity to ask questions, and I am satisfied with the answers I have received.
- I understand that I will be randomly allocated to one of four possible treatment groups, and that neither I nor the trial coordinator will know which group I am in.
- I voluntarily consent to take part in this research project, and understand that I can withdraw at any time without prejudice.
- I understand that my data will be stored for 15 years following the completion of the study, after which time it will be destroyed.
- I understand that this project has been approved by Curtin University Human Research Ethics Committee, and will be carried out in line with the National Statement on Ethical Conduct in Human Research (2007).
- I have received a copy of this Information Statement and Consent Form.

| **Participant Name** |  |
| --- | --- |
| **Participant Signature** |  |
| **Date** |  |

Declaration by researcher: I have supplied an Information Letter and Consent Form to the participant who has signed above, and believe that they understand the purpose, extent and possible risks of their involvement in this project.

| **Researcher Name** |  |
| --- | --- |
| **Researcher Signature** |  |
| **Date** |  |

Note: All parties signing the Consent Form must date their own signature.

**Appendix 2**

**Daily Migraine Diary**

| **Patient ID:** | - **Date**: |
| --- | --- |
| **Did you have a migraine today?** Yes  No   - If **NO**, please leave the remaining of today’s diary blank. - If **YES**, please answer the questions below. | |
| - **Frequency**: How many migraine attacks did you have today, separated by 2 hours each? | |
| **Severity:** On average, how severe was (were) the migraine(s)? The following line represents increasing pain intensity from ‘no pain’ to ‘worst possible pain’. Place a mark on the line that you feel best describes your level of pain.   \|  \| **No Pain Worst**  **Possible**  **Pain** \| Score in mm  (Investigator’s use only) \| \| --- \| --- \| --- \| | |
| Did you take anything to help relieve the pain? Yes  No  If YES, please list all medications, including over-the-counter medications.   \| **Medication name** \| **Number of tablets taken** \| \| --- \| --- \| \|  \|  \| \|  \|  \| \|  \|  \| | |
|  | |
| BEFORE YOUR MIGRAINE BEGAN, did you experience any of the following? Select all that apply.   \| Changes in vision \| Changes in smell \| Changes in sound \| Change in taste \| \| --- \| --- \| --- \| --- \| \| Excessive sleepiness \| Trouble talking \| Confusion \| Nausea (feeling sick) \| \| Vomiting \| Anxiety/irritability \| Low mood \| Neck ache \| \| Unsure \| None listed \|  \|  \|   BEFORE YOUR MIGRAINE BEGAN, did you have numbness or tingling in your face or arms?  Yes  No  Unsure  BEFORE AND/OR DURING YOUR MIGRAINE, did you experience any of the following sensations? Select all that apply.   \| Throbbing \| Shooting \| Stabbing \| Sharp \| \| --- \| --- \| --- \| --- \| \| Cramping \| Gnawing \| Hot/burning \| Aching \| \| Heavy (like a weight) \| Tender \| Splitting \| Tiring/exhausting \| \| Sickening \| Fear-causing \| Punishing/cruel \|  \| | |

**Appendix 3**

**Weekly Migraine Summary**

| **Patient ID:** | **Week:** |
| --- | --- |
| **Did you have a migraine over the past 7 days?** Yes  No | |

**Short-Form McGill Pain Questionnaire**

**PLEASE DESCRIBE YOUR PAIN DURING THE LAST WEEK** (Check off one box per line.)

|  | None | Mild | Moderate | Severe |
| --- | --- | --- | --- | --- |
| 1. Throbbing | 0  | 1  | 2  | 3  |
| 2. Shooting | 0  | 1  | 2  | 3  |
| 3. Stabbing | 0  | 1  | 2  | 3  |
| 4. Sharp | 0  | 1  | 2  | 3  |
| 5. Cramping | 0  | 1  | 2  | 3  |
| 6. Gnawing | 0  | 1  | 2  | 3  |
| 7. Hot-burning | 0  | 1  | 2  | 3  |
| 8. Aching | 0  | 1  | 2  | 3  |
| 9. Heavy (like a weight) | 0  | 1  | 2  | 3  |
| 10. Tender | 0  | 1  | 2  | 3  |
| 11. Splitting | 0  | 1  | 2  | 3  |
| 12. Tiring-Exhausting | 0  | 1  | 2  | 3  |
| 13. Sickening | 0  | 1  | 2  | 3  |
| 14. Fear-causing | 0  | 1  | 2  | 3  |
| 15. Punishing-Cruel | 0  | 1  | 2  | 3  |

**PLEASE RATE YOUR PAIN DURING THE LAST WEEK**

The following line represents pain of increasing intensity from “no pain” to “worst possible pain”. Place _a vertical mark (|) across the line in the position that best describes your pain **during the last week.**

| **No Pain** | **Worst Possible Pain** | Score in mm  (Investigator’s use only) |
| --- | --- | --- |

**CURRENT PAIN INTENSITY**

0  No pain

1  Mild

2  Discomforting

3  Distressing

Questionnaire developed by: Ronald Melzack

4  Horrible

5  Excruciating
